# Beta-blocker/ACE inhibitor therapy differentially impacts the steady state signaling landscape of failing and non-failing hearts

**DOI:** 10.1038/s41598-022-08534-0

**Published:** 2022-03-19

**Authors:** Andrea Sorrentino, Navratan Bagwan, Nora Linscheid, Pi C. Poulsen, Konstantin Kahnert, Morten B. Thomsen, Mario Delmar, Alicia Lundby

**Affiliations:** 1 Department of Biomedical Sciences, Faculty of Health and Medical Sciences, University of Copenhagen, Copenhagen N, Denmark; 2 Leon H Charney Division of Cardiology, NYU School of Medicine, New York, NY USA; 3 The Novo Nordisk Foundation Center for Protein Research, Faculty of Health and Medical Sciences, University of Copenhagen, Copenhagen N, Denmark

**Keywords:** Heart failure, Proteomics

## Abstract

Heart failure is a multifactorial disease that affects an estimated 38 million people worldwide. Current pharmacotherapy of heart failure with reduced ejection fraction (HFrEF) includes combination therapy with angiotensin-converting enzyme inhibitors (ACEi) and β-adrenergic receptor blockers (β-AR blockers), a therapy also used as treatment for non-cardiac conditions. Our knowledge of the molecular changes accompanying treatment with ACEi and β-AR blockers is limited. Here, we applied proteomics and phosphoproteomics approaches to profile the global changes in protein abundance and phosphorylation state in cardiac left ventricles consequent to combination therapy of β-AR blocker and ACE inhibitor in HFrEF and control hearts. The phosphorylation changes induced by treatment were profoundly different for failing than for non-failing hearts. HFrEF was characterized by profound downregulation of mitochondrial proteins coupled with derangement of β-adrenergic and pyruvate dehydrogenase signaling. Upon treatment, phosphorylation changes consequent to HFrEF were reversed. In control hearts, treatment mainly led to downregulation of canonical PKA signaling. The observation of divergent signaling outcomes depending on disease state underscores the importance of evaluating drug effects within the context of the specific conditions present in the recipient heart.

## Introduction

Heart failure (HF) is a multifactorial disease with a complex etiology and prognosis that affects an estimated 38 million people worldwide^1^. The incidence of HF continues to escalate as risk factors such as diabetes, obesity and ageing^1–3^ remain prevalent. HF can be classified based on whether or not ejection faction (EF) is preserved^4–8^. The present study focuses on the case of HF with reduced ejection fraction (HFrEF), a condition associated not only with a structural and mechanical deficit but also with an increased likelihood for the occurrence of life-threatening arrhythmias^7,9^.

Despite years of research, the molecular underpinnings of HFrEF remain unclear, due in part to the multifactorial nature of the condition. As the heart becomes unable to maintain normal output, a global remodeling of the contractile, metabolic and electrical functions takes place. A number of studies have addressed individual aspects of the phenotype, and in doing so, have advanced the field^10–12^. Yet, a fundamental knowledge gap remains regarding the global molecular profile of the failing heart, captured from samples treated under uniform conditions. Different studies have characterized transcriptomic changes in the process of heart failure^13,14^, but the conversion of transcriptomic data to protein function is challenging, given the poor correlation that exists between the transcriptome and the proteome of the same organ^15,16^. Proteomics studies such as those reported by Lau^17^ and Kuzmanov^18,19^ and colleagues have contributed to our understanding of the global changes in protein expression in animal models presenting cardiac remodeling and dysfunction. However, we still have limited knowledge of the protein- and signaling regulation characterizing a chronic state of heart failure, as well as the molecular changes that result, at the proteomics and phosphoproteomics level, following medical treatment. Current pharmacotherapy of HFrEF often includes a combination of angiotensin-converting enzyme inhibitors (ACEi) and β-adrenergic receptor blockers (β-AR blockers)^7,9,20,21^. The functional consequences of these drugs are well characterized, and some of their therapeutic effects are known to be extra-cardiac. Importantly, these drugs are also used for conditions in the non-failing heart (e.g., hypertension) or that primarily result from dysfunction in other systems (e.g., chronic kidney disease^22,23^). Yet, though the target receptors are abundantly present in the adult cardiac myocytes, the nature and the extent of changes caused in the molecular landscape of the heart as a result of chronic exposure to these drugs, are poorly characterized.

In this study, we have analyzed the proteome and the phosphoproteome of failing hearts. As an experimental model, HFrEF was induced in murine hearts by partial banding of the transverse aorta (transverse aortic constriction, or TAC). Data were also obtained from animals after two weeks of treatment with enalapril and metoprolol (an ACEi and a β-AR blocker, respectively). Herein, we provide a comprehensive characterization of the molecular profile of the failing hearts with and without treatment, and the first characterization of the consequences that these drugs have on non-failing hearts.

## Methods

### Surgical procedure and experimental protocol

A detailed description of the experimental protocol can be found in the online supplement. All experiments conformed to the EU directive (2010/63/EU) for animal research and were carried out under a license approved by the Animal Experiments Inspectorate. The study was carried out in compliance with the ARRIVE guidelines.

For induction of heart failure, C57BL6 male mice were anesthetized, their hearts exposed through lateral thoracotomy and a tight and permanent ligature applied to partially constrict the transverse aorta (TAC). Animals were allowed to recover under observation, and their heart function was examined by echocardiography at the end of eight weeks post-surgery. Control animals (SHAM) underwent the same surgical procedure, though only a loose ligature around the aorta was placed. Both SHAM and TAC groups were then followed for an additional two weeks after implantation of osmotic pumps to deliver either vehicle (Vh; saline) or treatment (Tr), the latter a combination of enalapril (ACE inhibitor-administered at the dose of 5 mg/kg/day) and metoprolol succinate (specific and nonvasodilating ß-adrenergic receptor blocker-administered at the dose of 30 mg/kg/day). The chronic administration of the medication have been chosen based on previous animal studies^24,25^. Efficacy of the treatment was evaluated in pilot experiments by reduced activity of plasma angiotensin converting enzyme and decreased heart rate in mice receiving treatment (Tr) compared to those receiving saline (Vh). Two weeks after onset of treatment (a total of ten weeks after surgery), heart function was re-evaluated by echocardiography and the animals were euthanized. After sacrifice, the cardiac samples were deidentified and experiments and analysis were performed in a blinded-to treatment manner. A detailed description of the methods can be found in the online supplemental materials.

## Results

### In-depth proteomic and phosphoproteomic characterization of TAC-induced failing hearts

**Figure 1:**
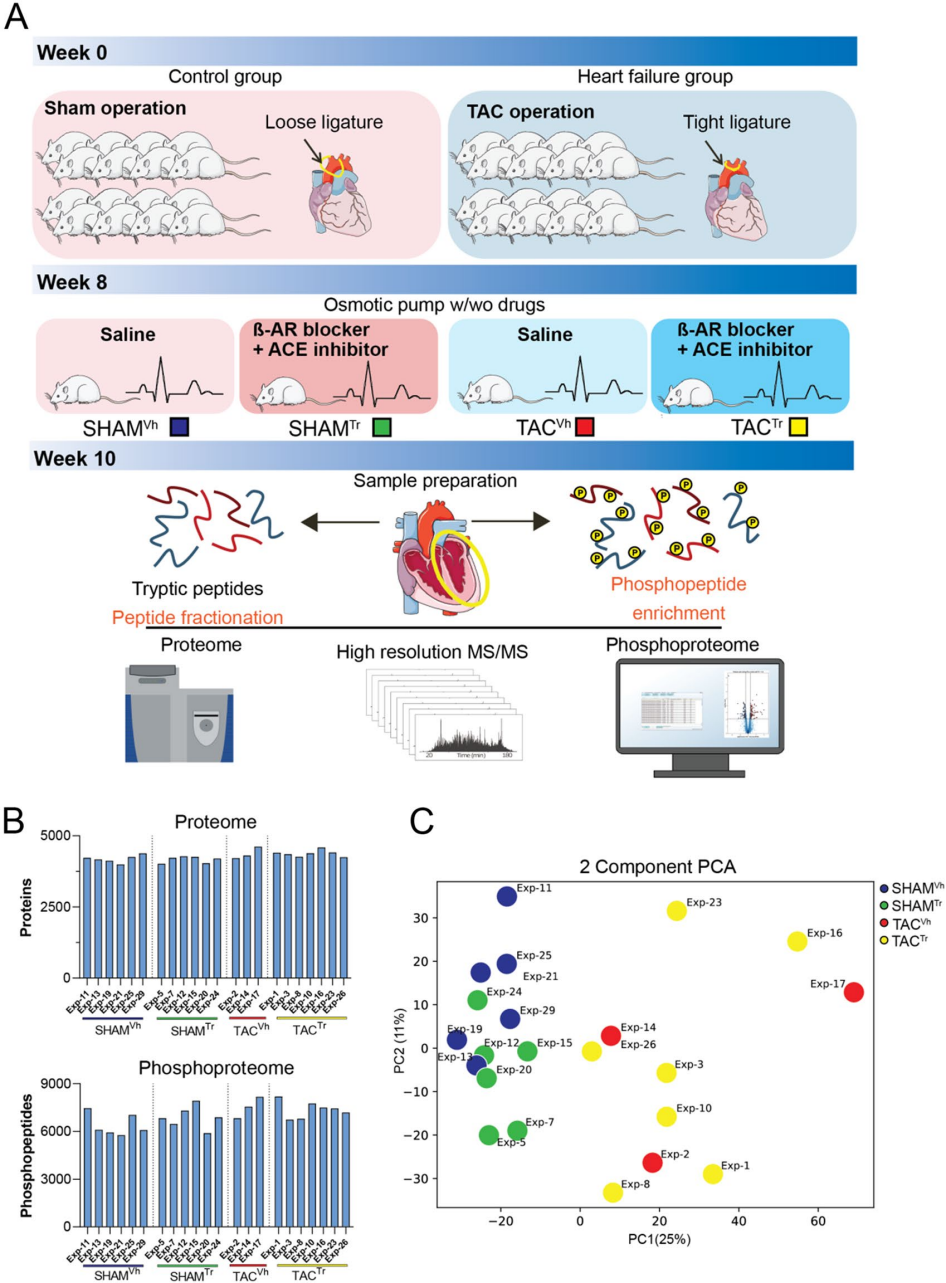
Experimental design and proteomics workflow. (**A**) Mice received either a loose ligature around the aorta (SHAM) or a tight ligature (TAC). Eight weeks post-surgery, cardiac function was evaluated by echocardiography and osmotic pumps delivering either saline (SHAM^Vh^ or TAC^Vh^) or ß-AR blocker and ACE inhibitor (SHAM^Tr^ and TAC^Tr^) were implanted. Ten weeks post-surgery, cardiac function was again evaluated by echocardiography, mice were sacrificed, hearts explanted, and the left ventricle dissected. Proteins were extracted from left ventricular tissues and subjected to deep proteome and phosphoproteome measurements by LC–MS/MS analysis. Heart and animal illustrations have been downloaded and modified from the open source Servier Medical Art (smart.servier.com) (**B**) Bar graphs summarizing the number of proteins (top) and phosphorylated peptides (bottom) measured for each heart sample. (**C**) Principal component analysis of measured protein intensities for all analyzed heart samples. Data from the four animal groups are illustrated by colors: data from animals in the SHAM^Vh^ group are depicted in blue, SHAM^Tr^ in green, TAC^Vh^ in red and TAC^Tr^ in yellow.

We measured the proteome and phosphoproteome of left ventricular tissue from mice that developed HFrEF as a result of transverse aortic constriction (TAC). Our studies sought to obtain a comprehensive perspective of the mechanisms underlying heart failure and the molecular pathways targeted by current treatment strategies. Fourteen and seventeen animals were subjected to the SHAM and the TAC procedures, respectively. A total of twelve SHAM and ten TAC animals completed the experimental protocol and were included in the final proteomic and phosphoproteomic evaluation (Fig. 1A). Echocardiography-based measurements of heart function prior to implantation of the osmotic pumps (8 weeks post-surgery) and at the end of treatment (10 weeks post-surgery) segregated by groups, and are presented in Table S1. As expected, TAC hearts presented with a significant elevation of left ventricular end systolic volume and reduced left ventricular ejection fraction when compared to SHAM controls. Left ventricular tissues from these animals were subjected to deep proteome and phosphoproteome measurements. We applied a label-free quantification approach by high-resolution liquid chromatography-mass spectrometry (LC–MS) performed on an Orbitrap mass spectrometer^26^. For phosphoproteomic analyses, peptides were enriched by titanium dioxide chromatography and analyzed in single-run format. For proteome analyses, peptide samples were pre-fractionated into 10 fractions prior to measurement^27,28^. Our experimental strategy enabled us to cover 6,004 cardiac proteins (Table S2) and 14,967 phosphorylation events (Table S3). The number of proteins and peptides with phosphorylation sites measured per heart sample are shown in Fig. 1B. Proteome and phosphoproteome measurements where highly reproducible with median Pearson correlation coefficients of biological replicates of 0.98 for the proteome and 0.86 for the phosphoproteome (Fig. S1A-B). As expected, correlations were in general higher for proteomic than for phosphoproteomic measurements. In agreement with previous phosphoproteomic investigations of tissue samples, the majority of phosphorylation events in the heart samples occurred on serine residues (86%), followed by threonine (13%), and to a much smaller extent tyrosine residues (1%) (Fig. S1C)^29–31^. Principal component analysis (PCA) of our proteomic data classified the heart samples according to their disease status (Fig. 1C). The drivers of the discrimination per disease status are shown in Fig. S11D. Cardiac contractile proteins were overrepresented in the hearts of SHAM animals, whereas TAC hearts were enriched for proteins grouped under cytoskeletal organization. Several proteomics studies have contributed to outlining molecular changes in heart failure, yet our dataset represents the most comprehensive proteomic and phosphoproteomic analysis of a failing heart with reduced EF to date (Fig. S2). The extent of the coverage and the quality of our data provided us with the foundation for an in-depth investigation of protein- and signaling changes under the experimental conditions tested.

### Proteome remodeling in left ventricles of TAC-induced failing murine hearts

**Figure 2:**
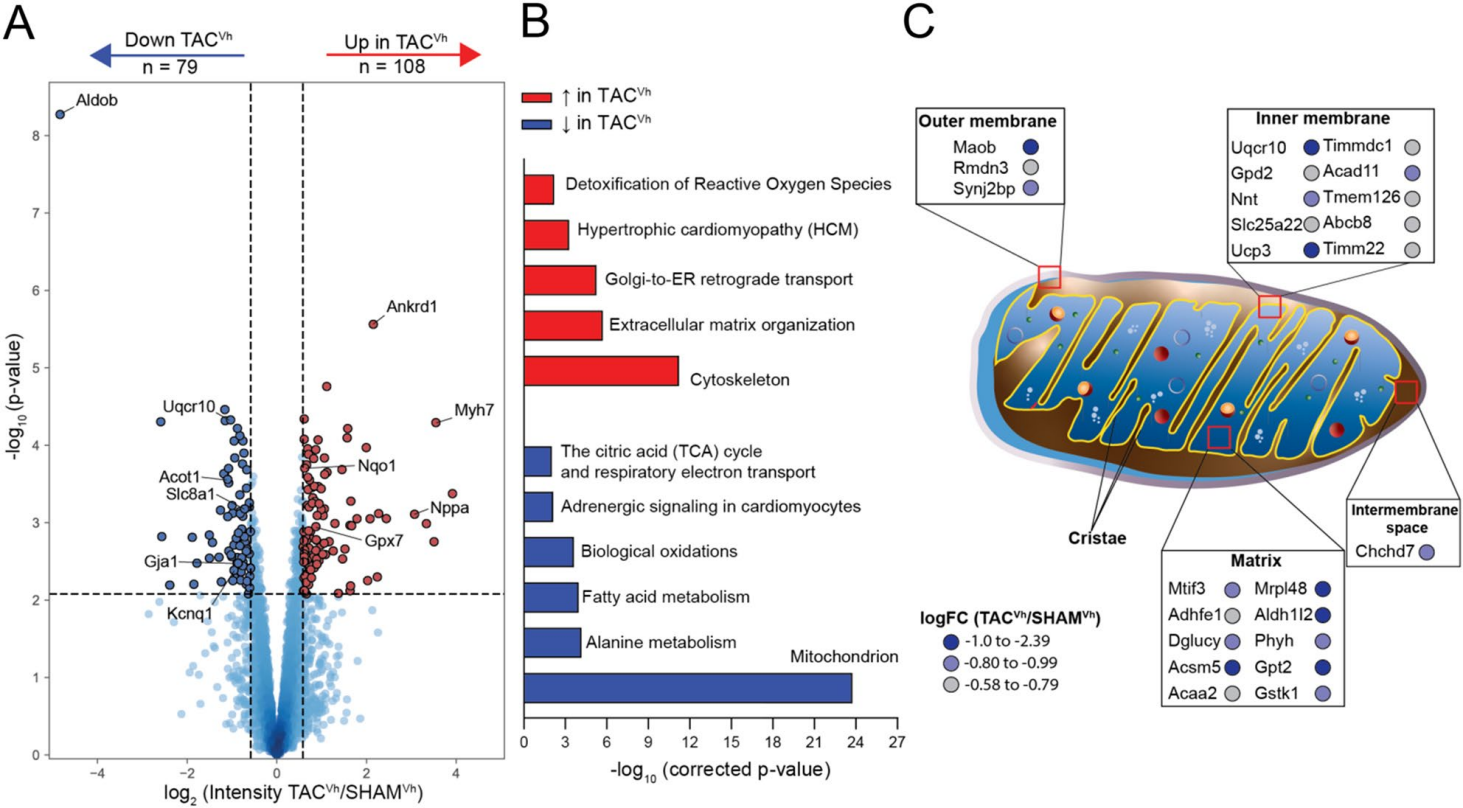
Downregulation of mitochondrial proteins in heart failure. (**A**) Volcano plot analysis of protein abundances in TAC^Vh^ mice compared to SHAM^Vh^ mice. Each point represents a protein, where statistical differences between TAC^Vh^ and SHAM^Vh^ animals were plotted as a function of the log2 transformed protein intensity ratios. Differentially expressed proteins are displayed as upregulated in red, and downregulated in blue. (**B**) Functional enrichment analysis of upregulated (red) and downregulated (blue) proteins in TAC^Vh^ mice compared to SHAM^Vh^ mice evaluated based on GO terms, Reactome and KEGG^68–70^ pathway analyses. (**C**) Visual representation of the downregulated proteins in TAC^Vh^ mice with mitochondrial localization.

We first focused our analyses on determining the changes in protein abundance recorded from left ventricular tissue associated with HFrEF after TAC. Accordingly, proteome data from TAC^Vh^ mice were compared to those obtained from SHAM^Vh^ mice. Quantitative proteomics analysis was based on label-free quantification (LFQ) intensities, which enabled us to evaluate relative abundances of 3,915 proteins for these animals (Table S4). Of these, 187 proteins were significantly differentially expressed (Fig. 2A): 79 proteins were downregulated and 108 proteins were upregulated as a consequence of heart failure.

Consistent with the functional findings, the significantly upregulated proteins included those previously characterized as upregulated in a failing heart, such as cardiac myosin heavy chain beta (Myh7), natriuretic peptide A (Nppa), and angiotensin converting enzyme (Ace)^32–36^. Functional enrichment analysis of all upregulated proteins grouped under terms consistent with TAC-induced remodeling, such as “extracellular matrix organization” and “hypertrophic cardiomyopathy” (Fig. 2B). The latter is coherent with the increased afterload imposed on the hearts and the resulting changes in left ventricular structure as detected by echocardiography (Tables S1).

Proteins downregulated in the failing hearts of TAC^Vh^ animals included several proteins involved in cell metabolism, such as fructose biphosphate aldolase B (Aldob), cytochrome b-c1 complex subunit 9 (Uqcr10), and acyl-coenzyme A thioesterase 1 (Acot1). Consistently, functional enrichment analysis revealed that downregulated proteins in the TAC^Vh^ model clustered around mitochondria and mitochondrial metabolic terms such as “citric acid cycle and respiratory electron transport.” (Fig. 2B). In fact, more than a third of the downregulated proteins reside in mitochondrial compartments, as evaluated by MitoCarta mapping (Fig. 2C). Taken together, these results yield a comprehensive picture of the specific mitochondrial proteins that are downregulated in TAC-induced HFrEF and that may be responsible for the metabolic dysfunction previously reported for this model^37,38^.The downregulation of proteins involved in mitochondrial function were accompanied by downregulation of proteins critical for maintenance of electrical cardiac function, such as the slow delayed rectifier K^+^ channel (Kcnq1), the inward rectifier K^+^ channel Kir2.1 (Kcnj11), the gap junction protein Connexin 43 (Gja1), the L-type Ca^2+^ channel (Cacna1c, Cacnab2) and the sodium/calcium exchanger NCX (Slc8a1). These data add to a body of evidence indicating electrical remodeling in HFrEF hearts^10,12^.

The functional enrichment also point to a downregulation of proteins involved in adrenergic signaling (Fig. 2B). This result aligns with previous data indicating beta-adrenergic desensitization in failing hearts^39^. Such signaling changes can better be evaluated from phosphoproteomics data and, accordingly, are discussed in more details below.

### Key signaling pathways are remodeled in left ventricle in TAC-induced HFrEF

**Figure 3:**
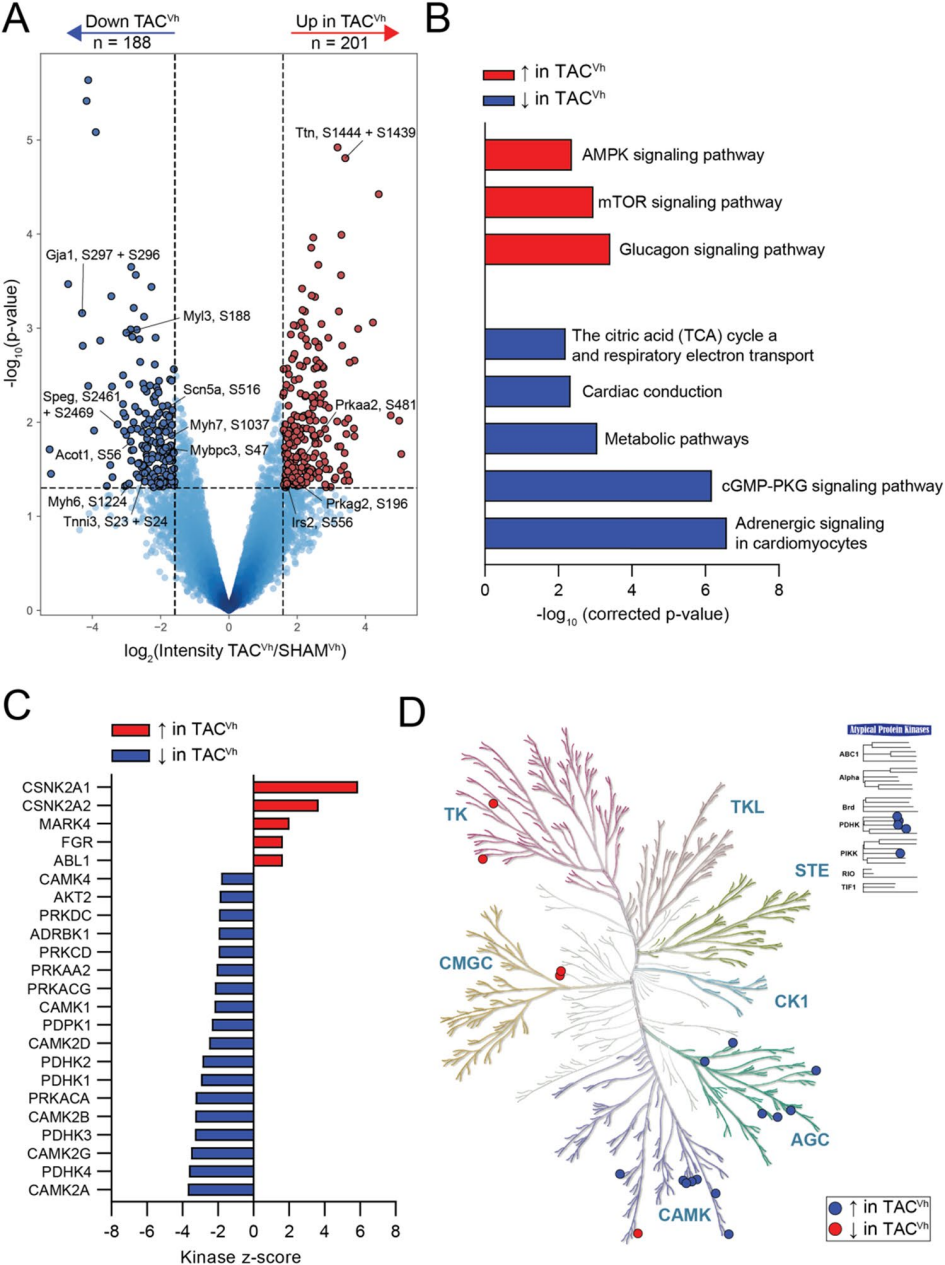
Phosphorylation mediated signaling changes in response to heart failure. Analyses of phosphorylated peptide intensities from TAC^Vh^ versus SHAM^Vh^ animals. (**A**) Volcano plot analysis showing phosphorylated peptides that are either downregulated (blue) or upregulated (red) in heart failure animals compared to controls. (**B**) Functional enrichment analysis of proteins with either upregulated (red) or downregulated (blue) phosphorylation sites in TAC^Vh^ compared to SHAM^Vh^ evaluated based on KEGG^68–70^ and REACTOME pathway analyses. (**C**) Kinase-Substrate Enrichment Analysis results that show kinases with either up- (red) or downregulated (blue) activity in the TAC^Vh^ hearts compared to SHAM^Vh^ hearts. (**D**) Phylogenetic tree of the kinome (KinMap^71^), showing kinases with upregulated (red) or downregulated (blue) activities in response to heart failure.

To investigate which signaling pathways contribute to functional remodeling in the heart failure model, we set out to quantify differences in protein phosphorylation between TAC^Vh^ and SHAM^Vh^ animals. We obtained quantitative information from 6547 phosphorylation events across heart samples from TAC^Vh^ and SHAM^Vh^ animals (Table S5, Fig. S3). Among these, 201 phosphorylated peptides were significantly upregulated and 188 phosphopeptides were significantly downregulated in TAC^Vh^ compared to SHAM^Vh^ controls (Fig. 3A).

Several upregulated phosphorylation sites were from proteins associated with metabolic remodeling, such as AMPK (Prkaa2^S481^, Prkag2^S196^), Irs2^S556^, and Camkk2^S495^. Proteins containing upregulated phosphorylation sites were functionally clustered in terms associated with metabolic processes (‘glucagon signaling pathways’, ‘mTOR signaling pathways’ and ‘AMPK signaling pathways’; see Fig. 3B). These data, together with the proteomics results, are consistent with the notion that the molecular machinery involved in cell metabolism is prominently regulated in HFrEF.

Many of the phosphorylation sites found to be downregulated in TAC^Vh^ animals reside on proteins involved in myofilament regulation (Myh6^S750, S881,S1039,S1224,S1309,S1337,S1467,S1478,S1609^, Myh7^S1037^, Mybpc3^S47^, Myl3^S188^, Speg^S2461,S2469^, Tnni3^S23,S24^) or electrical conduction (Gja1^S296,S297,S326,S328^, Scn5a^S516^). When grouped together by functional enrichment analysis, downregulated phosphorylation sites related to cardiac conduction, cell metabolism and adrenergic regulation (Fig. 3B). These findings go beyond the notion that a chronic increase in afterload impacts the electrical and metabolic homeostasis of the left ventricle; here, we specifically identify the individual phosphorylation sites that are regulated under these conditions.

The functional enrichment analysis highlights the biological functions of the proteins with altered phosphorylation sites. However, such analysis does not outline which kinases orchestrate the response. To address this, we used an integrative computational approach termed Kinase Substrate Enrichment Analysis^40^. KSEA estimates differential kinase activity based not on the abundance or phosphorylation state of the kinase itself, but on the collective phosphorylation changes of their identified substrates. For example, we found increased activity of casein kinase II (Csnk2a1, Csnk2a2), reflecting the increased phosphorylation of Hdac2^S394^, a site that has been associated with cardiac hypertrophy^41^ and increased phosphorylation of Ace^S1305^, which regulates retention of the enzyme in the plasma membrane^42^, among other targets (Table S6). Using this approach we found maladaptive activities in kinases of the AGC and CaMK superfamilies in heart failure (Fig. 3C-D, Table S6). Of particular relevance, the analysis showed reduced activity of Camk2 kinases (Camk2d, Camk2a, Camk2b, Camk2g) and protein kinase A (PKA; encoded by Prkaca). In the case of PKA and Camk2d we identified a reduction in the phosphorylation state of sites relevant to electrical function and intracellular calcium homeostasis. These include phospholamban phosphorylation sites Pln^S16,T17^ (key regulators of calcium reuptake in myocytes^43^), ryanodine receptor sites Ryr2^S1402, S2803, S2807^ (which modulate RyR2 channel open probability^44^), and ion channel sites Kcnq1^S481^ and Scn5a^S516^, which affect gating of their respective channels^45,46^. Of note, in the case of PKA as well as Camk2d, a change in the phosphorylation state of the kinases themselves was noted (residues Prkaca^T197^ and Camk2d^S319,T336,T337^) as well as reduced phosphorylation of the activation sites of phosphodiesterase 3A, Pde3a^S310,S435^, which indirectly opposes beta-adrenergic signaling pathways^47^. Our analysis also identified reduced activity of kinases that affect cell metabolism and in particular Pyruvate Dehydrogenase Kinases (Pdhk4, Pdhk3, Pdhk1, and Pdhk2). Specifically, we found reduction of substrates directly regulated by Pdhk4 such as Pdha1 at its regulatory sites S232, S293, S300, as well as of Sirt1^S14^ and Foxo1^S467^. Taken together, our observations suggest a signaling dysregulation of kinases implicated in β-adrenergic signaling response, as well as in mitochondrial metabolic remodeling.

### Signaling rewiring in TAC-induced HFrEF hearts following pharmacological treatment

**Figure 4:**
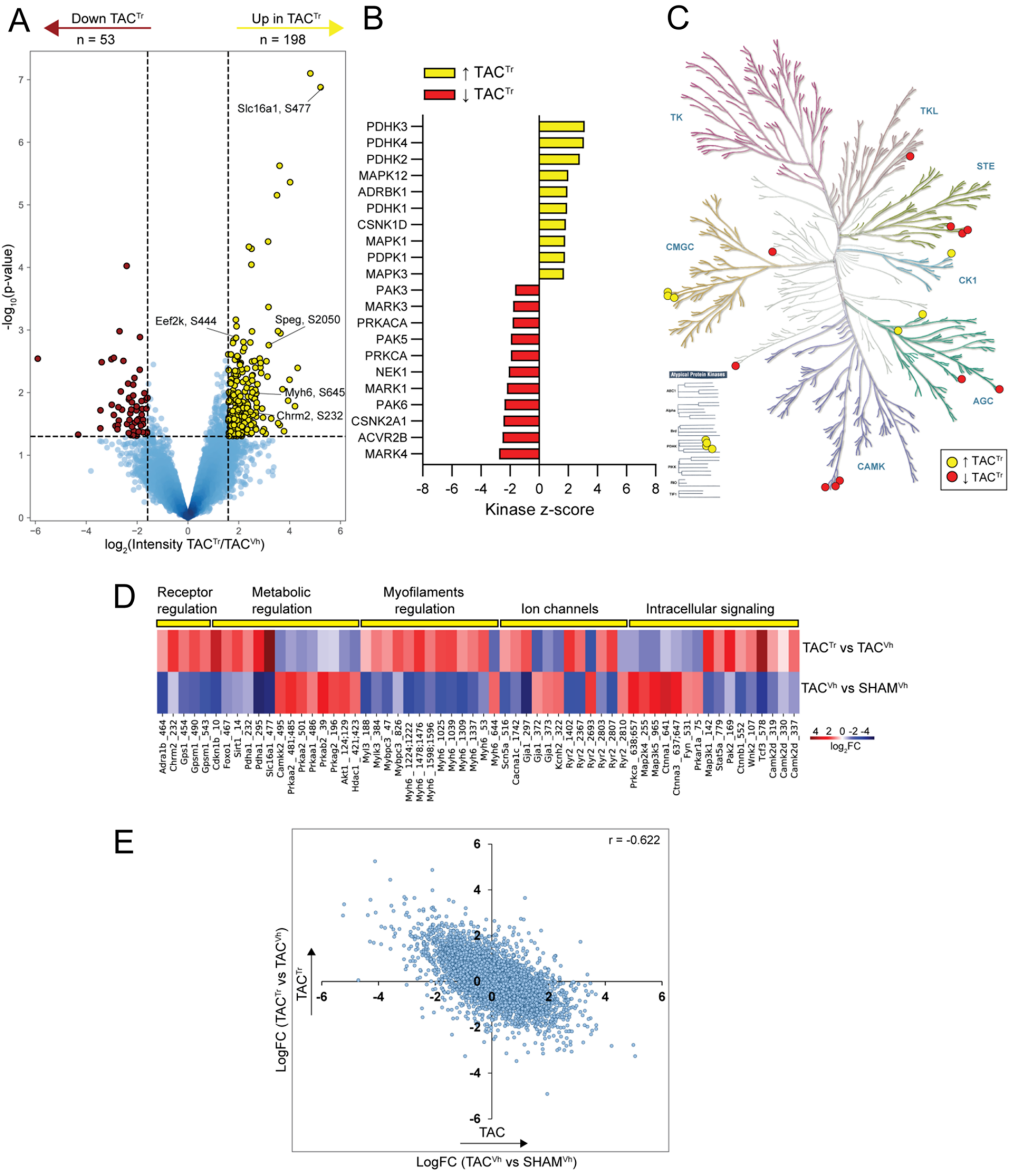
Phosphoproteome regulation in response to treatment in TAC animals. (**A**) Volcano plot analysis evaluating phosphorylation response in heart failure mice in response to drug treatment (TAC^Tr^ versus TAC^Vh^). The plot shows phosphorylated peptides that are downregulated (red) or upregulated (yellow) in animals with heart failure in response to drug treatment. (**B**) Kinase-Substrate Enrichment Analysis results that show kinases with either up- (yellow) or down regulated (red) activity in the TAC^Tr^ hearts compared to TAC^Vh^ hearts. (**C**) Phylogenetic tree of the kinome (KinMap^71^) visualizing kinases with upregulated (yellow) or downregulated (red) activities in heart failure animals upon drug treatment. (**D**) Heat map visualizing the fold-change in site-specific phosphorylation for select proteins with essential cardiac functions. The lower panel shows the fold-change between TAC^Vh^ and SHAM^Vh^. The upper panel shows the fold-change between TAC^Tr^ and TAC^Vh^. The phosphorylation change in response to TAC is reversed by the treatment. (**E**) Scatter plot of fold-changes for all measured phosphorylated peptides with the fold-change for TAC^Tr^/TAC^Vh^ shown as function of fold-change in TAC^Vh^/SHAM^Vh^. There is a negative correlation for the phosphorylation fold-changes. The Pearson correlation coefficient (r) is provided.

Standard treatment of HFrEF patients includes the use of ACEi and β-AR blockers (such as enalapril and metoprolol, respectively). Therefore, we sought to determine the effects of enalapril/metoprolol on the proteome and phosphoproteome of HFrEF hearts (TAC^Tr^ vs TAC^Vh^). Two weeks of treatment did not lead to significant changes in the cardiac proteome (Table S7). In contrast, 251 phosphorylation events were significantly affected: 198 phosphopeptides were significantly upregulated and 53 significantly downregulated (Figs. 4A, S4) in the treated hearts. The full list of regulated phosphorylation sites is provided in Table S8. Many upregulated phosphorylation sites reside on myofilament proteins, such as Myh6^S53,S645,S1039,S1476,S1478,S1552,T1607,S1609,S1632^, Myh7^S643, S1037,S1550^ and Mylk3^S306^. Functional enrichment analysis showed that the upregulated phosphorylation sites clustered under terms related to metabolic equilibrium (“AMPK signaling pathway”, “Insulin signaling pathway”, “Fatty acid biosynthesis”) and cardiac contraction (Fig. S4C). KSEA was used to identify, based on the phosphorylation abundance of their substrates, kinases with predicted increased or reduced activity in treated hearts (Fig. 4B, C). Increased activity was noted, among others, in pyruvate dehydrogenase kinases (Pdhk4, Pdhk3, Pdhk2, and Pdhk1), and in the beta-adrenergic receptor kinase 1 GRK2 (coded by gene Adrbk1). Of note, the activity of these kinases was decreased consequent to TAC. A reversal of their activity was therefore found with treatment. A similar switch was observed for casein kinase II; Cskn2a1 activity was increased consequent to TAC but reduced in response to treatment.

The reversal in kinase activities noted above extended to multiple components of the dataset. We focused on five functionally relevant categories: receptor regulation, metabolic regulation, myofilament regulation, ion channels, and intracellular signaling. The data are summarized in the heatmap shown in Fig. 4D. For each phosphorylation site noted in the bottom, the color indicates an increase (red) or a decrease (blue) in phosphorylation levels when comparing either TAC^Vh^ vs SHAM^Vh^ (bottom) or TAC^Tr^ vs TAC^Vh^ (top). Notice the multiple switches between red and blue across the dataset, indicating that treatment led to a reversal of the phosphorylation state of specific sites. Specific phosphosites that were downregulated as consequence of heart failure and then upregulated in response to treatment include Pdha1^S232, S295^, Foxo1^S467^, and Sirt1^S14^, mainly involved in metabolic remodeling. For receptor regulation we observed reduced phosphorylation of alpha-1-adrenergic receptor (Adra1b^S464^) and the muscarinic acetylcholine receptor M2 (Chrm2^S232^) in heart failure, which were reversed by treatment. Moreover, the enalapril-metoprolol combination brought about a switch in the representation of multiple RyR2 phosphorylation sites, some with known functional relevance (e.g., RyR2^S2810^), of Scn5a^S516^ and of Gja1^S297, S372,S373^. Finally, notice the treatment-induced shift in phosphorylation sites on key mediators of intracellular signaling pathways including Camk2d^S319,S330,S337^, Prkca^S638,S657^, Map2k4^S255^ and Map3k5^S965^. The switch in phosphorylation state was not limited to the examples illustrated in the heatmap. We evaluated the change in phosphorylation state across all measured phosphorylated peptides and observed that they were inversely correlated with a Pearson correlation coefficient of − 0.6 (Fig. 4E). That is, the overall phosphorylation changes consequent to TAC were reversed by treatment. In summary, our data show that combination therapy with a β-AR blocker and an ACEi induces a switch in the activity of multiple kinase signaling pathways affected by TAC-induced HF, beyond those well known as directly initiated by the specific transmembrane receptors.

### Effect of enalapril and metoprolol on the phosphoproteome of hearts in the SHAM group

**Figure 5:**
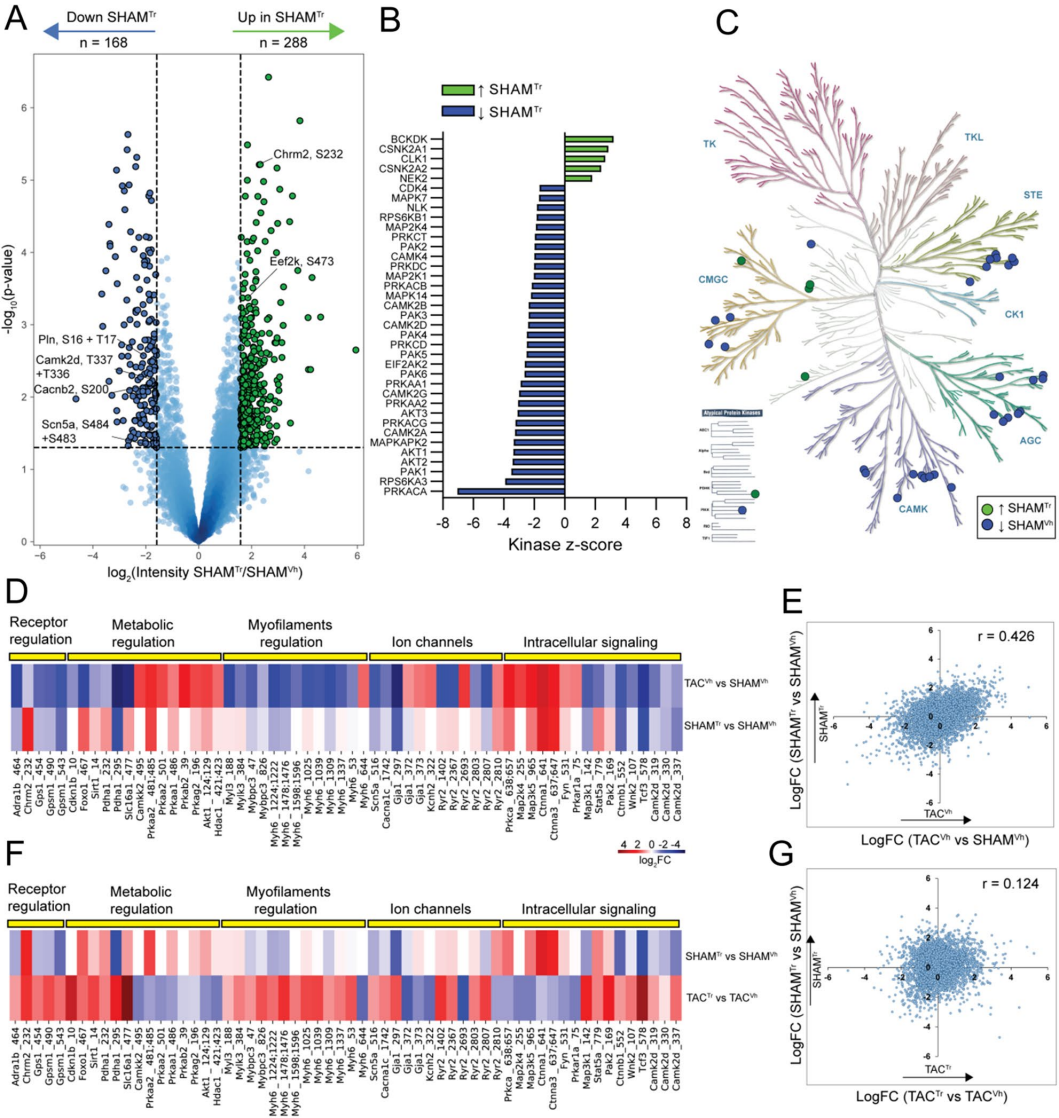
Phosphoproteome regulation in response to treatment in control animals differs from response in TAC animals. (**A**) Volcano plot analysis evaluating which phosphorylated peptides are downregulated (blue) and which are upregulated (green) in control animals exposed to treatment with β-blocker and ACE inhibitor (SHAM^Tr^ versus SHAM^Vh^). (**B**) Kinase-Substrate Enrichment Analysis results that show kinases with either up- (green) or down regulated (blue) activity in the SHAM^Tr^ hearts compared to SHAM^Vh^ hearts. (**C**) Phylogenetic tree of the kinome (KinMap^71^) showing kinases with increased (green) or decreased (blue) activities in control animals in response to drug treatment. (**D-F**) Heat map representing the fold-change in protein phosphorylation for selected phosphorylation sites. Each site is described by gene name and amino acid position of phosphorylation underneath the heat map. (**D**) The phosphorylation fold-change is shown for control animals in response to treatment (bottom, SHAM^Tr^/SHAM^Vh^) or in response to heart failure (top, TAC^Vh^/SHAM^Vh^). (**E**) Scatter plot of fold-changes for all measured phosphorylated peptides, where the fold-change in the comparison SHAM^Tr^/SHAM^Vh^ is shown as function of the fold-change for the comparison TAC^Vh^/SHAM^Vh^. r is the Pearson correlation coefficient. Phosphorylation changes induced by TAC^Vh^ correlate positively with phosphorylation changes induced by SHAM^Tr^ (**F**) The phosphorylation fold-change is shown for failing- (bottom, TAC^Tr^/TAC^Vh^) and control (top, SHAM^Tr^/SHAM^Vh^) hearts in response to treatment. (**G**) Scatter plot of fold-changes for all measured phosphorylated peptides in SHAM^Tr^/SHAM^Vh^ shown as function of the fold-change in TAC^Tr^/TAC^Vh^. r is the Pearson correlation coefficient. The effect of the treatment on phosphorylation state is not correlated between TAC and SHAM mice, suggesting a systematic differences in the effect of treatment in failing and non-failing heart.

The combination of ACEi and β-AR blocker treatment is not only used in cases of demonstrated HFrEF, but in other conditions that can benefit from the extra-cardiac effects of these drugs^22,23,48,49^. As a first approach to characterize the molecular consequences of treatment in a non-failing heart, we analyzed the proteome and phosphoproteome from left ventricles of SHAM animals receiving two weeks of treatment with either ACEi and β-AR blocker (SHAM^Tr^) or placebo (SHAM^Vh^). As in the case of failing hearts, treatment did not significantly change left ventricular protein expression (Table S9). In contrast, there was a large impact on phosphorylation mediated signaling. We observed upregulated phosphorylation of Ace^S1305^ (regulated by CK2) and phosphorylation on the beta-adrenergic receptor Adrb1^S401,S417^ (involved in the recruitment of the B-arrestin 2)^42,50^. Quantitative analysis of 6,353 phosphorylated peptides identified 168 phosphorylation events that were significantly downregulated, and 288 phosphorylation events that were significantly upregulated, in the treated animals (Figs. 5A, S4, Table S10). Upregulated sites included Gravin^S18,S22,S767^, HDAC2^S394^ and Chrm2^S232^. Downregulated sites included multiple ion channels (Scn5a^S483,S484^, Kcnh2^S1140^, Kcnq1^T481^, Kcnj3^S442^, Cacnb2^S200,S510,S524^, Kcnd2^S574^), molecules involved in intracellular calcium homeostasis (Pln^S16,T17^), and key regulators of intracellular signaling (Camk2d^T337^, Pde3a^S310,S438^, Pde4d^S150^, Akt1^S129^, Gsk3a^S21^, Gsk3b^S9^). Reduced phosphorylation levels at these sites reflect a general decrease in phosphorylation of downstream targets of the β-adrenergic signaling pathway, as expected for exposure to metoprolol. Consistent with the latter, functional enrichment analysis of proteins with downregulated phosphorylation sites highlighted terms related to β-adrenergic signaling (“adrenergic signaling in cardiomyocytes”, “calcium signaling pathway,” “cAMP signaling pathway,” “insulin signaling pathway” and “MAPK signaling pathway;” Fig. S4F), and KSEA analysis showed a prominent effect on kinases of the AGC, CamK and STE superfamilies (Fig. 5B,C), including PKA and all Camk2 kinases (Camk2d, Camk2a, Camk2b, and Camk2g) as well as Akt and Pak kinases.

Previous studies have shown that pressure overload-induced heart failure leads to a desensitization of βAR-initiated pathways^51,52^. Consistent with this, we found that multiple phosphorylation sites modified in HFrEF were also modified, in the same direction, after administration of ACEi and β-AR blocker in SHAM animals (Fig. 5D). Notice in particular the consistencies in the changes in the “intracellular signaling” and the “receptor regulation” groups. This phosphorylation change in similar direction for HFrEF and treated SHAM animals was a general trend, as illustrated by a correlation coefficient of 0.4 when comparing phosphorylation changes in response to heart failure to the phosphorylation change in control animals in response to treatment for all phosphorylated peptides (Fig. 5E). Interestingly, given that the phosphorylation state of SHAM and TAC hearts differ, the impact of treatment on phosphorylation mediated signaling also differed substantially between the groups (Fig. 5F,G). In fact, the effect of treatment on phosphorylation sites grouped under ‘receptor regulation’ and ‘intracellular signaling’ was opposite for SHAM hearts to that observed when the same treatment was administered to TAC hearts (Fig. 5F). The extent of the different phosphorylation outcome in TAC hearts compared to SHAM hearts in response to treatment is illustrated in Fig. 5G. When analyzing all phosphorylated peptides, there was no correlation between the changes observed in TAC hearts to those in SHAM hearts in response to treatment. That is, the signaling response in the heart elicited by combination therapy of ACEi and β-AR blocker depends on the disease state of the heart. The response in a diseased heart cannot be extrapolated to an expected response in a healthy heart.

## Discussion

In the work presented here we explored the proteome and phosphoproteome changes associated with a model of heart failure, and define for the first time global changes resulting from treatment with β-AR blocker (metoprolol) and ACE inhibitor (enalapril) in HFrEF hearts as well as in control hearts.

Our results showed that the predominant effect on the proteome of the left ventricle of TAC-induced HFrEF is a marked metabolic remodeling, intertwined with changes in proteins relevant to the electrical and mechanical functions of the heart. The metabolic changes parallel similar findings previously reported in heart failure in humans^44^ and reflect the metabolic flexibility and stress responses of cardiac cells as they remodel consequent to the excessive afterload (e.g. downregulation of Acot1, Ehhadh, Acaa2 and upregulation of Nqo1, Gpx7). Our results also confirm reduction in proteins relevant to electromechanical coupling in HFrEF hearts, such as the gap junction protein Connexin43 (Gja1), which can predispose to arrhythmic events^53–55^.

The primary findings in our study come from analysis of the signaling changes evaluated by phosphoproteomic analyses. Previous studies have identified several members of the AGC and Camk superfamilies as maladaptive in heart failure^56^. Herein, analysis of phosphorylated substrates pointed to reduced activities of protein kinase A (Prkaca) and the Camk2 family. Of the latter, Camk2d is the predominant cardiac isoform and we observed reduced phosphorylation of this kinase at residues 319, 330 and 337. Additionally, also targets directly regulated by Camk2d had reduced phosphorylation levels^45,57^, such as Pln^T17^and Scn5a^S516^. The reported findings overall suggest that reduced Camk2d signaling cascades, and not exclusively upregulation, may contribute to electrical instability and heart failure.

Our observation of reduced activity of protein kinase A was, among others, linked to reduced phosphorylation at Pln^S16^ and Tnni3^S23,S24^ (Troponin I) in HFrEF animals^60,61^. PKA is known as the main kinase downstream of β-adrenergic receptor activation, and the downregulation of PKA activity suggested by the phosphoproteomic data support the notion that the cardiac dysfunction in the TAC model is characterized by an uncoupling between β-adrenergic responses and calcium/myofilaments mechanisms.

An important new finding resulting from our work relates to the effect that treatment with a β-AR blocker and an ACE inhibitor has on the phosphorylation state of proteins depending on whether they are captured from a failing heart or from a control. Phosphoproteomic analysis of treated SHAM hearts revealed a clear downregulation of canonical targets regulated by PKA signaling (e.g. Pln^S16^), consistent with known cardiac effects of the β-AR blocker. Secondary to the reduction in PKA signaling we observed reductions in Camk2 and Akt2 activities. The reduction in Camk2d activity and downstream targets (e.g. Pln^T17^) are in line with previous findings from our group^26^ and supports the notion that β-blockade can exert its effects not only by reducing PKA signaling pathways but also by downregulating Camk2d signaling. The treatment also led to a predicted decrease in Akt2 activity; Akt2 is an important regulatory isoform involved in insulin signaling. A cross-talk between the beta-adrenergic and insulin signaling pathways in hearts has been reported^59,62,63^, and here our findings suggest that β-blockade can directly attenuate insulin signaling, affecting cardiac metabolism and glucose uptake.

In failing hearts, treatment led to a markedly different signaling outcome than in the control hearts. The most striking difference was that our phosphoproteomic data suggest a reversal of the Pdhk4 signaling pathways upon treatment in failing hearts. In particular the phosphoproteome data highlighted the upregulation of pyruvate dehydrogenase E1 component subunit alpha (Pdha1^S232, S293, S300^)^58^, which is a direct target of the pyruvate dehydrogenase kinase family and implicated in the regulation of the glucose oxidation^64–66^. This finding may suggest a potential restored metabolic flux. We observed that a number of phosphorylation changes consequent to TAC were reversed by combination therapy. This reversal was not confined to specific categories (as highlighted in Fig. 4D) but also present across the phosphoproteome (as shown in Fig. 4E). We speculate that this reversal in the phosphorylation state of hundreds of proteins signals the ability of treatment to bring the function of the myocyte closer to the control (“healthy”) state. Yet, whether this reversal is a mechanism by which treatment improves function, or it is a passive, secondary consequence of the improved function brought about via a separate mechanism, remains to be defined.

We made the observation that there is partial correlation between the phosphorylation change induced by HFrEF and the change induced by treatment in SHAM animals. This effect may be attributable, among other things, to the convergence of beta-AR desensitization known to occur in the failing hearts, and the loss of beta-AR signaling expected from the use of a beta-AR blocker (metoprolol in our case). Furthermore, we found a lack of correlation between the change in the phosphoproteome caused by the treatment of failing hearts (i.e., comparison TAC^Vh^ vs TAC^Tr^) and the change caused by the same treatment in SHAM animals (SHAM^Vh^ vs SHAM^Tr^). The combination treatment examined in this study is commonly used both in patients exhibiting HFrEF and also in those with functionally competent hearts. Our paradoxical observation highlights the importance of evaluating possible drug effects within the context of the specific conditions present in the recipient heart.

In summary, we have used state-of-the-art proteomics and phosphoproteomics to elucidate protein abundance and phosphorylation signaling modulated in the heart consequent to TAC-induced HFrEF, with or without combination therapy. Our data coverage extended beyond that achieved by previous studies, providing confirmation of previous results and also unveiling novel molecular signatures. We provide the first global analysis of the changes in protein abundance and phosphorylation state of the left ventricle that result from a combination therapy of common clinical use. Our data show that treatment reverses specific elements of the metabolic, electrical and signal remodeling that results from TAC. The phosphorylation changes consequent to combination therapy were profoundly different for failing and non-failing hearts. Overall, our study extends the molecular catalog of changes brought about by pressure overload, identifies novel modulatory pathways, and stresses the molecular/functional differences that can result from these specific therapeutic agents depending on the functional state of the receiving hearts.

### Limitations of the study

The present study provides an extensive investigation of the molecular mechanisms regulated in an animal model of heart failure and by the clinical treatment. In the interpretation of the results, it is important to take into account that our treatment has direct effects on the heart and in addition have peripheral effects on blood pressure, water balance and electrolytes that will affect the physiology of the heart. In the present study, we did not quantify or control these parameters. Hence, additional functional studies related to the effect of the medications on the blood pressure are required in future.

An additional important challenge to be considered for the interpretation of our data is that we used the kinase substrate enrichment analysis (KSEA) to predict the kinases regulated in the cardiac phenotype and by the treatment. On one hand, the computational analysis of the kinases based on the phosphoproteomics data led to the identification of new and well-known dysregulated kinases, but on the other hand it exclusively suggested and not functionally defined the kinase activity. Therefore, additional functional follow-up are required to integrate our results.

Given the clear limitations of our experimental model along with inference of the kinase activity, extrapolation from our results to the clinically relevant environment can be only speculative and needs to be done with caution.

## Supplementary Information

Supplementary Information 1.

Supplementary Information 2.

Supplementary Information 3.

Supplementary Information 4.

Supplementary Information 5.

Supplementary Information 6.

Supplementary Information 7.

Supplementary Information 8.

Supplementary Information 9.

Supplementary Information 10.

Supplementary Information 11.

## Data Availability

The mass spectrometry proteomics data have been deposited to the ProteomeXchange Consortium via the PRIDE^67^ partner repository with the dataset identifier PXD024525. The data are accessible through https://www.ebi.ac.uk/pride/archive and project name ‘Beta-blocker/ACE inhibitor therapy differentially impacts the steady state signaling landscape of failing and non-failing hearts’.
