# Supplementary material for: Beta-blocker/ACE inhibitor therapy differentially impacts the steady state signaling landscape of failing and non-failing hearts
Source: Sci Rep. 2022 Mar 19;12:4760. doi: 10.1038/s41598-022-08534-0 (PMC8934364; doi:10.1038/s41598-022-08534-0)
Supplement: Supplementary file 1 — Supplementary Information 1. [file 41598_2022_8534_MOESM1_ESM.docx]

**Supporting Information**

**Contents**

**Materials and Methods**

Heart failure induction, treatments, and statistical analysis…………………….…….………....1

Mass spectrometry sample preparation…………………………………………………………….....2

Mass spectrometry measurements …………...…………………………………………….……...…3

Mass spectrometry data analysis………………………….…………………… ………………….....3

Functional enrichment………………..……………………………………………………………....4

KSEA analysis……………………………………………………………………………………......4

KinMap analysis……………………………………………………………………………………...4

MitoCarta analysis…………………………………………………………………………………....4

Data availability………………………………………………………………………………………......4

**Supplementary Figures**

Legends for the supplementary figure S1-S6…………………………………………………...........6

**Supplementary Tables**

Legends for the supplementary table S1-S10……………………………………………………..…11

**References**...……………………………………………………………………………………………..12

**Materials and Methods**

**Heart failure induction, measurements and treatments**

All experiments conformed to the EU directive (2010/63/EU) for animal research and were carried out under a license approved by the Animal Experiments Inspectorate. The study was carried out in compliance with the ARRIVE guidelines. For induction of heart failure, 31 male C57BL6 mice (Taconic, DK) were housed at 22°C with a 12h light/dark schedule and were provided food and tap water *ad libitum*. At 5 weeks of age, the mice were anesthetized (2% isoflurane in O_2_) and randomized and their hearts exposed through lateral thoracotomy to transverse aortic constriction (TAC, n=17) or sham operations (n=14), as previously described ^25^. We anticipated a 20% higher mortality in the TAC operated group, so this group size was 3 animals larger. Carprofen (5 mg kg^−1^, once daily for 3 days) and buprenorphine (0.05 mg kg^−1^, thrice daily) were used as postoperative analgesia. Eight weeks after surgery, all mice received an implantable osmotic pump (Alzet, model 2002) delivering 0.5 µl/h s.c. for 14 days. TAC or sham-operated mice were randomized to receive saline or a combination of enalapril (administered at the dose of 5mg/kg/day) and metropolol (administered at the dose of 30mg/kg/day) in saline. The chronic administration of the medication have been chosen based on previous animal studies. ^6, 30^. At weeks 8 and 10, we performed ultrasound echocardiographic evaluation of ventricular size and cardiac function during isoflurane anesthesia (2%), as previously described ^27^. We measured heart rate, fractional shortening, left ventricular volume, and ejection fraction, calculated from LV inner diameters using an algorithm recommended by the manufacturer. Immediately after the echocardiographic study at 10 weeks, mice were sacrificed, the hearts excised, the LVs dissected out and frozen by placing them in liquid N_2_. A total of thirteen SHAM and eleven TAC animals completed the experimental protocol.

Efficacy of the enalapril treatment was verified in pilot experiments by measuring plasma ACE activity levels in a group of control animals with or without treatments (enalapril + metoprolol) by using the ACE kit (Trinity Biotech). ACE levels in control animals were 114±1.49 U/l, whereas in control treated mice were 46±12.91 U/l (p-value = 1.02E-03 vs control, unpaired student t-test). Statistical analysis of the physiological data was performed with Graph Prism (version 8.4.3-GraphPad Software, Inc.). The data were tested for normality (Shapiro-Wilk test) for the assignment to parametric or non-parametric. When the null hypothesis of normality was met, unpaired student’s t-test was used. When null hypothesis of normality was rejected, non-parametric Mann-Whitney test was performed. The data are presented as mean ±SEM.

**Mass spectrometry sample preparation**

The sample preparation was performed as described previously ^19, 20^.Frozen left ventricles from individual mice were homogenized separately using Precellys 24 (Bertin Instruments) in ice-cold lysis buffer supplemented with protease and phosphatase inhibitors (5mM EDTA, 150mM NaCl, 10mM KCl, Complete protease inhibitor cocktail tablet Roche, 50 mM Tris (pH 8.5), 1 mM sodium orthovanadate, 5 mM sodium fluoride, 5 mM b-glycerophosphate). Samples were incubated rotating head-over-tail at 4°C for 2 hours before centrifugation at 15,000g for 20 min at 4°C. Proteins were precipitated by adding four volumes of ice cold acetone and protein concentrations were measured (Quick Start Bradford Dye Reagent X1, Bio-Rad). Proteins were resuspended in Gnd-HCl buffer (6M Gnd-HCl, Complete protease inhibitor cocktail tablet Roche, 50 mM Tris (pH 8.5), 1 mM sodium orthovanadate, 5 mM sodium fluoride, 5 mM b-glycerophosphate ) and reduced and alkylated (5mM TCEP, 10mM chloroacetamide) in darkness for 15min before being digested with endoproteinase Lys-C (Wako) at an enzyme to protein ratio of 1:100 for 1 hour at 30C followed by dilution (1:12 with 50 mM Tris-HCl pH 8) and digestion with trypsin (16 h) at 37 °C, 750 rpm (Life technologies, USA, 1:100 enzyme:protein ratio). Trypsin digestion was quenched by lowering the pH to ~2 with trifluoroacetic acid (TFA). Samples were centrifuged at 16,000g for 20 min, and supernatants were desalted and concentrated on Sep-Pack C_18_ cartridges (Waters). Peptides were eluted with 40% acetonitrile (ACN) followed by 60% ACN, and organic solvents subsequently evaporated by vacuum centrifugation. Peptide concentrations were measured and 500 µg peptide was taken aside for proteome measurements and the remainder was used for phosphopeptide enrichments. Peptide samples for proteome measurements were fractionated at high pH on an Ultimate3000 system (Thermo) as previously described ^16^. In brief, peptides were fractionated by micro-flow reverse-phase ultra-high pressure liquid chromatograpy (UPLC) on an Dionex UltiMate 3000 UPLC system (Thermo Scientific, USA) equipped with an ACQUITY UPLC CSH C_18_ Column (130Å, 1.7 µm, 1 mm x 150 mm) at 30 μL/min flow rate. The following 85 min gradient elution programme was used employing a binary pump connected to Solvent A (5 mM ammonium bicarbonate (ABC), pH 8) and B (100% ACN): 0–50 min: 4.5–22.5% B, 50–55 min: 22.5–63% B, 55–60 min: 63% B isocratic, 60–62 min: 63–81% B, 62–70 min: 81% B isocratic, 70–75 min: 81–4.5% B, followed by column re-equilibration at 4.5% B for 10 min. Outflow from 4–60 min was collected in 1-min intervals into 12 concatenated fractions in the autosampler. Fractions were acidified by addition of 5 μL 5% formic acid and fraction volume was reduced by vacuum centrifugation. If not specified otherwise, chemicals and reagents were acquired from Sigma-Aldrich, USA. Chromatography solvents were acquired from VWR, USA. Phosphopeptides were enriched with TiO_2_ beads essentially as described previously ^19, 21^. Three milligram of TiO2 beads (GL Sciences Inc.) suspended in 10 µl 2,5-dihydroxybenzoic acid (DHB) [0.02 g DHB/ml 80% ACN, 0.5% acetic acid (AcOH)] were used per sample. Beads were pre-incubated with mixing before being added to each of the samples, which were then incubated with gentle rotation for 30 min at 30 rpm. The TiO_2_ beads were quickly spun down, and the supernatants were removed. The beads were washed with 80% Acetonitrile, 6% TFA and transferred onto in-house packed C_8_ stage-tips. While on the coloumn, beads were washed with first 80% Acetonitrile, 6% TFA, followed by 50% Acetonitrile, 6% TFA, then 10% Acetonitrile, 6% TFA and lastly 80% Acetonitrile, 6% TFA. Samples were eluted with 20 ul of 5% NH4OH followed by 20μL 10% NH4OH, 25% ACN. Ammonia and organic solvents were evaporated with a vacuum centrifuge. The peptides were then acidified in 1% TFA, 5% ACN and loaded onto in-house packed C_18_ stage-tips, where they were stored until measurement.

**Mass spectrometry measurements**

Peptides were eluted and brought to a concentration of 0.2 µg/µL (diluted in 5% ACN, 0.1% TFA) in 96-well microtiter plates and analyzed by online reversed-phase liquid chromatography (nanoflow Easy-nLC system, Proxeon Biosystems, Odense, Denmark) coupled to a Q-Exactive HF quadrupole Orbitrap tandem mass spectrometer (Thermo Electron, Bremen, Germany). Proteome peptide samples were separated on 15 cm fused-silica emitter columns (75 μm inner diameter) pulled and packed in-house with reversed-phase ReproSil-Pur C_18_-AQ 1.9 μm resin (Dr. Maisch GmbH, Ammerbuch-Entringen, Germany) in a 64 min multi-step linear gradient (0.1% formic acid constant; 5–25%ACN in 45 min, 25–45%ACN in 8 min, 45–80% ACN in 2 min) followed by short column re-equilibration (80–5%ACN in 7 min, 5%ACN for 2 min). Full-MS spectra (375–1500 m/z) were acquired after accumulation of 3,000,000 ions in the Orbitrap (maximum fill time 25 ms) at 120,000 resolution. A data-dependent Top12 method enabling deep proteome measurements was used, sequentially isolating the most intense precursor ions (up to 12 per full scan) for higher-energy collisional dissociation (HCD) in an octopole collision cell. MS/MS spectra of fragment ions were recorded at resolution of 30,000 after accumulation of 100,000 ions in the Orbitrap (maximum fill time of 45 ms). For the phosphopeptide-enriched samples, peptides were separated by a linear gradient of ACN in 0.5% AcOH for 265min in a 50-cm fused-silica emitter in-house packed with reversed-phase ReproSil-Pur C_18_-AQ 3 mm resin (Dr. Maisch GmbH) (0.1% formic acid constant; 5-25%ACN in 220 min, 25-60% ACN in 30 min, followed by a short column re-equilibration 60-5% ACN in 10 min and 5%ACN for 5 min). Column effluent was directly ionised in a nano-electrospray ionisation source operated in positive ionisation mode and electrosprayed into the mass spectrometer. For the phosphopetide-enriched samples, Full-MS spectra (300–1750 m/z) were acquired after accumulation of 1,000,000 ions in the Orbitrap (maximum fill time 20 ms) at 120,000 resolution. A data-dependent Top10 method enabling deep phosphoproteome measurements was used, sequentially isolating the most intense precursor ions (up to 10 per full scan) for higher-energy collisional dissociation (HCD) in an octopole collision cell. MS/MS spectra of fragment ions were recorded at resolution of 60,000 after accumulation of 200,000 ions in the Orbitrap (maximum fill time of 108 ms).

**Mass spectrometry data analysis**

Raw MS data was processed using the MaxQuant software ^2^ (Max-Planck Institute of Biochemistry, Department of Proteomics and Signal Transduction, Munich) and proteins identified with the built-in Andromeda search engine by searching MS/MS spectra against an in-silico tryptic digest of a database containing all reviewed SwissProt mouse protein entries (downloaded on 05/03/2020). For the proteome dataset the MS/MS spectra were searched with Carbamidomethyl-Cysteine as fixed modification, as well as oxidation (M), acetylation of protein N-termini whereas for the phospho-proteome dataset in addition to fixed modification, phospho (STY) as variable modifications was used. Due to the similarity of the samples the match-between-runs option was enabled with default parameters. The minimum peptide length was set to seven amino acids (default) peptide mass tolerance was set to 10 ppm and fragment mass tolerant to 0.02 Da. False-discovery rate cut-offs were set to 1 % on peptide and protein level, only allowing high-quality identifications to pass. All peptides were used for protein quantification. Animal inclusion criteria in the final proteomic and phosphoproteomics evaluation were that the ejection fraction at the time point of implanting osmotic mini-pumps were within two standard deviations of the average of the group. For two animals, the phosphoproteomics experiments failed technically leading to identification of only 2719 (SHAM^Vh^) and 344 (TAC^Tr^) phosphorylated peptides, respectively. Those animals were accordingly removed form subsequent analysis.

All data were filtered for decoy and contaminant proteins. For the phospho-proteomic dataset, intensities were log2 transformed and median normalized using an in-house python script. For the proteomic dataset log2 transformed LFQ intensities were used, and therefore no normalization was performed.

Proteins where no intensities was recorded in any of the samples were removed from the analysis. Post filtering the dataset, remaining missing values were imputed using downshift imputation method where values were drawn from a distribution with a width 0.03 and a downshift 1.8 compared to the original sample distribution.

**Quantitative analysis**

Global analysis of significant differences between the TAC and SHAM groups was done separately in proteome and phosphor-proteome datasets. Prior to comparative analyses Principal Component Analysis (PCA) was performed. PCA analysis was used to identify outliers that were subsequently removed prior to protein expression analysis. Differential protein expression was calculated using the R package LIMMA^8^ based empirical Bayes moderated test statistics. Proteins were deemed regulated if their intensity comparisons between groups had a P-value < 0.05 and a fold-change greater than 1.5 (log2 scale = 0.5849). For phospho peptide a P-value cut-off of 0.05 and fold-change greater than 3 (log2 scale = 1.5849) was used. These cut-offs were applied in all volcano plots. Proteins and peptides identified as significantly differentially regulated were used for further analysis, such as functional enrichment analysis.

**Functional enrichment analysis**

Functional enrichment analysis of significantly differentially expressed proteins and phosphosites was carried out (based on their gene names) using g:Profiler ^24^(<https://biit.cs.ut.ee/gprofiler/gost>) and KEGG pathways analysis ^10, 11, 9^. Proteins with positive and negative fold change were analyzed separately.  Mus musculus and all known genes were selected as a parameter for the final analysis. Highly enriched terms are filtered and plotted as a bar plot using adjusted P-value at a cut-off of 0.01.

**KSEA analysis**

Kinase Substrate Enrichment Analysis was conducted using KSEA App^29^ (<https://casecpb.shinyapps.io/ksea/>). The analysis was performed by using all mono-phosphorylated peptides as input. PhosphoSitePlus + NetworKIN was selected as kinase-substrate dataset and default settings were used for the remaining parameters.

**KinMap**

Mouse gene names were converted to their human orthologs by using an ensembl biomart ortholog mapping database (downloaded 24.11.2020, instruction can be found here: <http://www.ensembl.info/2009/01/21/how-to-get-all-the-orthologous-genes-between-two-species/>).

Human ortholog genes were then filtered for kinases based on a list of all human kinases from KinHub (<http://www.kinhub.org/kinases.html>). The resulting kinases were mapped onto the human kinome tree using KinMap^4^ (<http://www.kinhub.org/kinmap/>).

**MitoCarta analysis**

To localize the mitochondrial gene in cell, the MitoCarta3.0^23^ mouse inventory database was used, which is a collection of more than 1000 nuclear and mitochondrial genes encoding proteins with strong support of mitochondrial localization and sub-mitochondrial compartment.

**Data Availability Statement**: The mass spectrometry proteomics data have been deposited to the ProteomeXchange Consortium via the PRIDE^22^ partner repository with the dataset identifier PXD024525. The data are accessible through <https://www.ebi.ac.uk/pride/archive> and project name ‘Beta-blocker/ACE inhibitor therapy differentially impacts the steady state signaling landscape of failing and non-failing hearts’.

**Supplementary Figures**


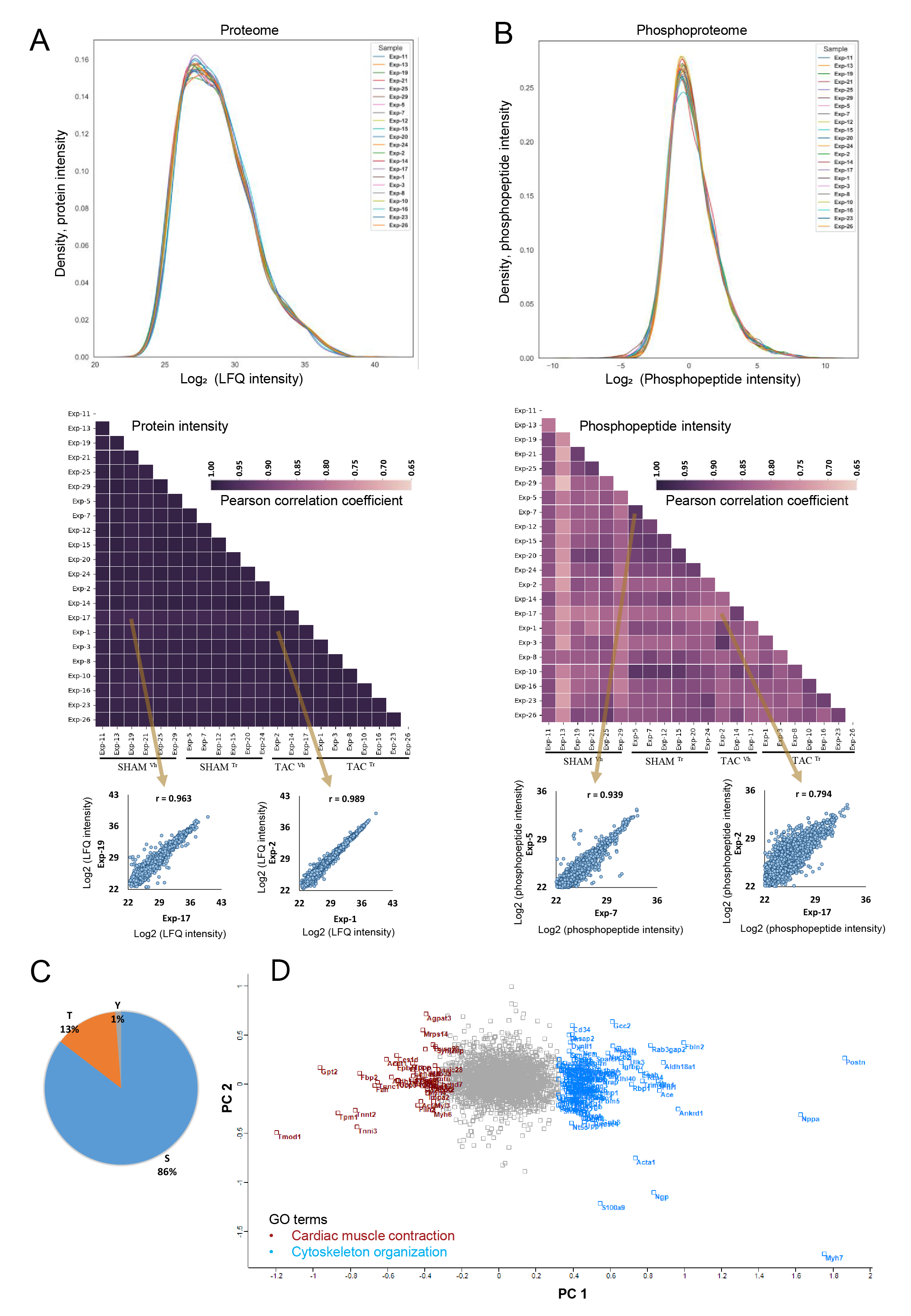


*Supplemental Figure 1. Quality control evaluation of proteome and phosphoproteome data for all animals in the study***.** (**A**) Density plot of log_2_-transformed LFQ protein intensities (top) and Pearson correlation coefficient for the proteome of the experimental groups (bottom). The smaller inset below the correlation heatmap show examples of the correlations between protein intensities of two experiments represented in the heatmap. (**B**) Density plot of log_2_-transformed median normalized intensities of phosphopeptides (top) and Pearson correlation coefficient between phosphopeptide intensities measured across samples (bottom). The inset figures show examples of the correlation between peptide intensities from two experiments. (**C**) Pie chart representing the percentage of amino acids found to be modified by phosphorylation in this study. (**D**) Loading covariances for the principal component analysis shown in Figure 1D of the proteomic data. The proteins driving the separation along the first component were evaluated by gene ontology (GO) functional enrichment. Proteins included in the GO term analysis representing SHAM animals are depicted in red. Proteins included in the GO term evaluation of proteins representing TAC animals are depicted in blue.

| **Proteome in animal models of cardiac dysfunction and heart failure** | | | | | | |
| --- | --- | --- | --- | --- | --- | --- |
| ***Species*** | ***Study*** | ***Year*** | ***Proteins identified*** | ***Model*** | ***Tissue*** | ***Ref*** |
| Mouse | This study |  | 6,004 | TAC-pressure overload | Heart-Left ventricle |  |
| Mouse | Kuzmanov et al. | 2020 | 3,630 | TAC-pressure overload | Whole heart | ^13^ |
| Mouse | Lu et al. | 2019 | 1,638 | Ischemic/Dilated cardiomyopathy | Whole heart | ^18^ |
| Mouse | Lau et al. | 2018 | 8,064 | Hypertrophic model | Whole heart | ^14^ |
| Mouse | Kuzmanov et al. | 2016 | 3,580 | Dilated cardiomyopathy | Whole heart | ^12^ |
| Mouse | Dao-Fu Dai et al. | 2013 | 961 | TAC-pressure overload | Heart-Left ventricle | ^3^ |
| ***Other species*** | | | | | | |
| Human | Kuzmanov et al. | 2021 | 4,185 | Patients with HCM | Heart septum | ^13^ |
| Pig | Xue Wang et al. | 2020 | 2,099 | Ischemic myocardial stunning | Heart-Left ventricle | ^28^ |
| Human | Liu et al. | 2019 | 4,263 | Dilated cardiomyopathy | Heart-Left ventricle | ^17^ |
| Rat | Jia et al. | 2019 | 1,290 | Myocardial infarction | Whole heart | ^7^ |
| Pig | Gedik | 2017 | 3,674 | Coronary occlusion/reperfusion | Heart-Left ventricle | ^5^ |
| Human | Gedik | 2017 | 3,390 | Surgical coronary revascularization | Human-left ventricle | ^5^ |

| **Phosphoproteome in animal models of cardiac dysfunction and in heart failure** | | | | | | |
| --- | --- | --- | --- | --- | --- | --- |
| ***Species*** | ***Study*** | ***Year*** | ***Phosphopeptides identified*** | ***Model*** | ***Tissue*** | ***Ref*** |
| Mouse | This study |  | 14,967 | TAC-pressure overload | Heart-Left ventricle |  |
| Mouse | Kuzmanov et al. | 2020 | 4,642 | TAC-pressure overload | Whole heart | ^13^ |
| Mouse | Kuzmanov et al. | 2016 | 3,908 | Dilated cardiomyopathy | Whole heart | ^12^ |
| Mouse | Su et al. | 2014 | 1,724 | Chronic renal failure | Heart-Left ventricle | ^26^ |
| Mouse | Yu-Wang Chang et al. | 2013 | 918 | TAC-pressure overload | Heart-Left ventricle | ^1^ |
| ***Other species*** | | | | | | |
| Pig | Xue Wang et al. | 2020 | 4,332 | Ischemic myocardial stunning | Heart-Left ventricle | ^28^ |
| Pig | Ledee et al. | 2016 | 1,896 | Ischemic reperfusion | Heart-Left ventricle | ^15^ |

*Supplemental Figure 2. Summary of studies analyzing cardiac proteomes and phosphoproteomes in models of cardiac dysfunction and heart failure***.** Studies of cardiac proteomes are shown in the top table, studies of cardiac phosphoproteomes are shown in the bottom table. For each study, the species investigated as well as the cardiac disease model is provided. In the top model the number of proteins is provided and in the bottom table the number of phosphopeptides is provided. This table does not claim completeness.


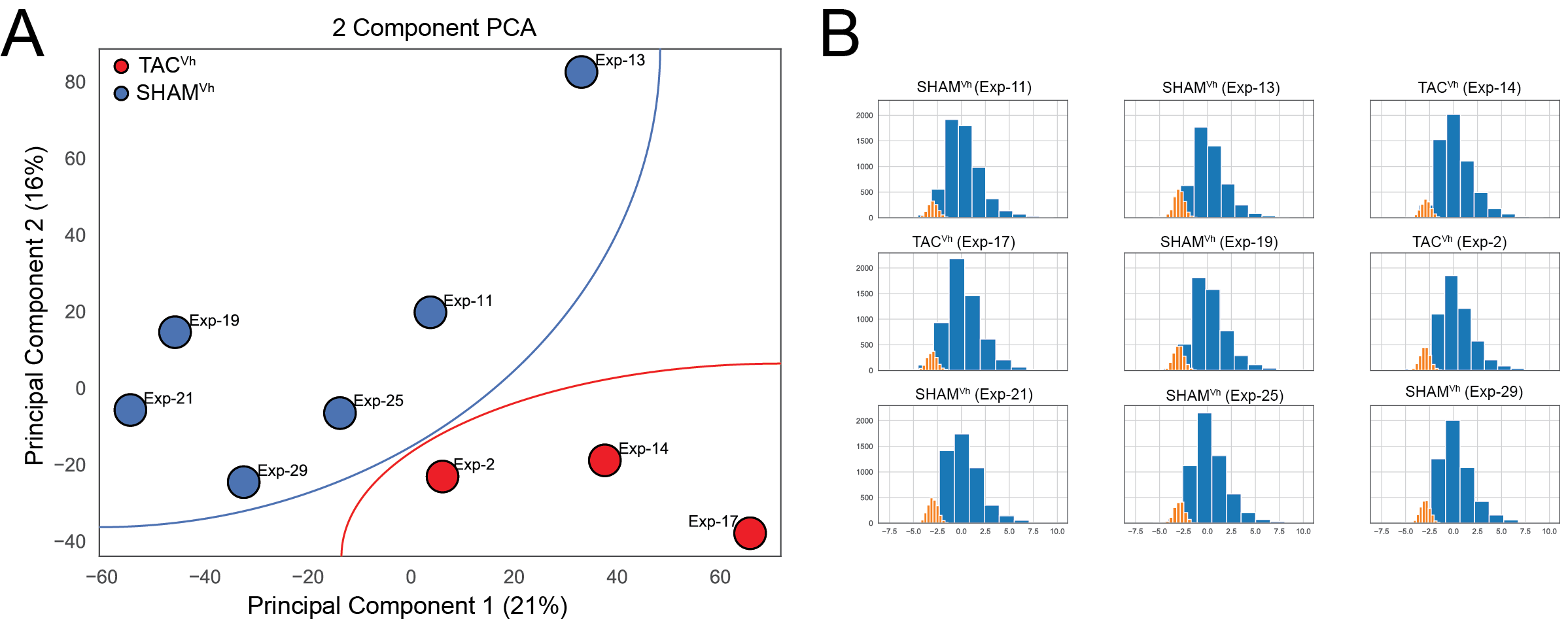


*Supplemental Figure 3. Quality control evaluation of phosphoproteome data for SHAM^Vh^ and TAC^Vh^ animals***.** (**A**) Principal component analysis (PCA) of measured phosphopeptide intensities for SHAM^Vh^ and TAC^Vh^ animals. (**B**) Histogram distributions of all phosphopeptide intensities per animal. Measured intensities are in dark blue. Imputed values are shown in orange.

*
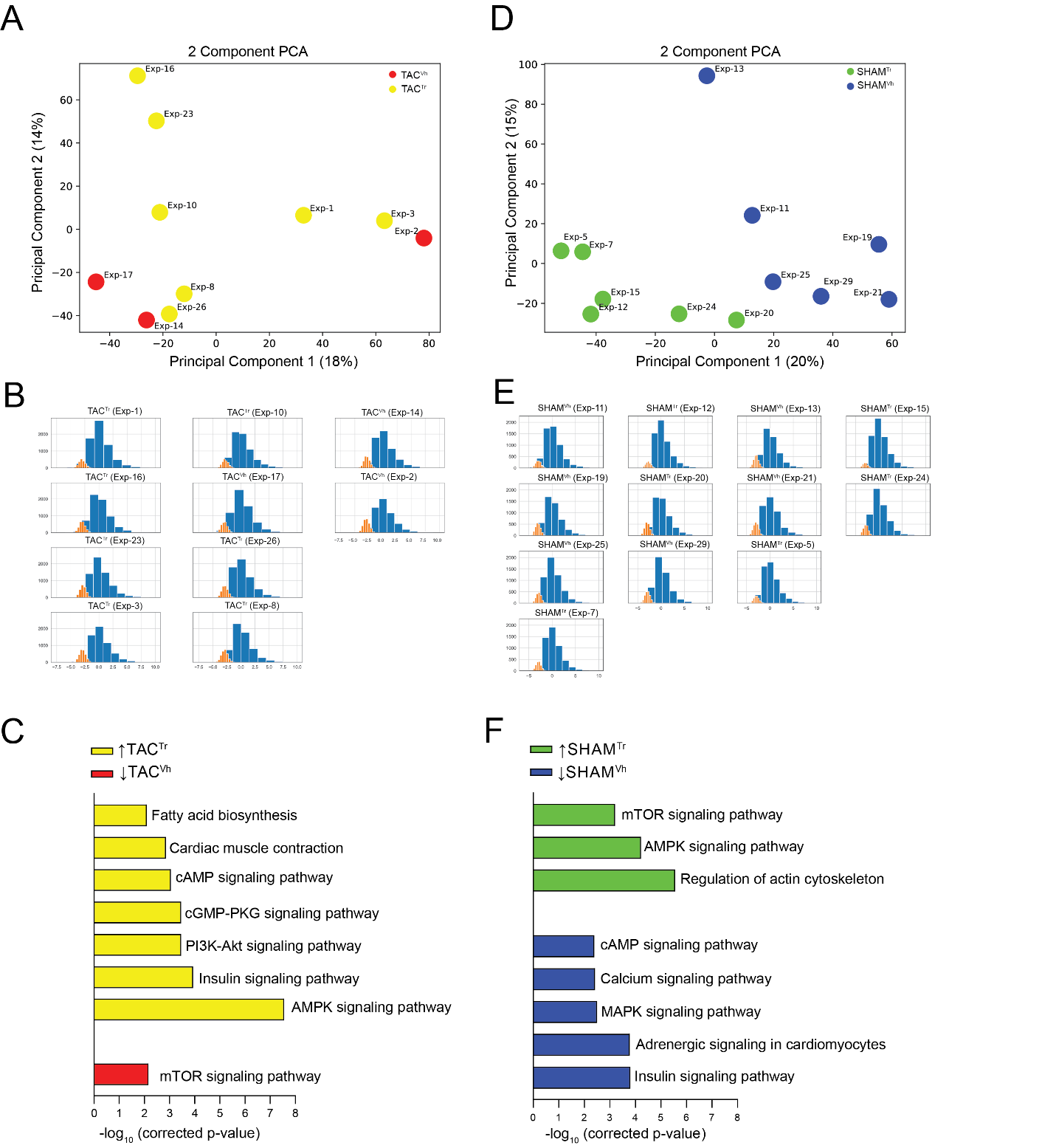
*

*Supplemental Figure 4. Quality control evaluation of phosphoproteome data for SHAM and TAC animals with or without treatment***.** (**A**) Principal component analysis (PCA) of measured phosphopeptide intensities for TAC^Vh^ and TAC^Tr^ animals. (**B**) Histogram distributions of phosphopeptide intensities per animal. Measured intensities are in dark blue. Imputed values used in quantitative analyses are shown in orange. **(C)** Functional enrichment analysis of proteins with phosphorylation sites that are upregulated (yellow) or downregulated (red) in response to treatment as evaluated by KEGG pathway analyses. (**D**) Principal component analysis (PCA) of measured phosphopeptide intensities for SHAM^Vh^ and SHAM^Tr^ animals included in the quantitative analysis (**E**) Histogram distributions of phosphopeptide intensities per animal. Measured intensities are in dark blue. Imputed values used in quantitative analyses are shown in orange. **(F)** Functional enrichment analysis of proteins with phosphorylation sites that are upregulated (green) or downregulated (blue) in response to treatment as evaluated by KEGG pathway analyses.

**Supplementary Tables**

**Table S1:** Echocardiographic measurements at 8 and 10 weeks post-surgery in SHAM and TAC animals**.** Values are reported as mean ±SEM. The ‘Phenotype 8 weeks post-surgery’ table contains echocardiographic measurements after 8 weeks post-surgery in the SHAM and TAC group. The ‘Phenotype 10 weeks post-surgery’ table contains echocardiographic measurements after 10 weeks post-surgery in the SHAM^Vh^ and TAC^Vh^ group. The ‘Phenotype 10 weeks post-surgery + treatments’ table contains echocardiographic measurements 10 weeks post-surgery in the SHAM^Tr^ and TAC^Tr^ group. The ‘SHAM 10 weeks after surgery (vehicle vs treatment)’ table contains comparison between SHAM^Tr^ and SHAM^Vh^. The ‘TAC 10 weeks after surgery (vehicle vs Treatment)’ table contains a comparison between TAC^Tr^ and TAC^Vh^ animals. The data were tested for normality (Shapiro-Wilk test) for the assignment to parametric or non-parametric tests. When the null hypothesis of normality was met, unpaired student’s t-test was used. When null hypothesis of normality was rejected, non-parametric Mann-Whitney test was performed. The data are presented as mean ±SEM.

**Table S2:** List of all identified proteins. The first sheet represents the list of all identified proteins, whereas the second one contains explanations for the column headers.

**Table S3:** List of all identified phosphorylated peptides. The first sheet represents the list of all identified phosphorylated peptides, whereas the second one contains explanations for the column headers.

**Table S4:** List of the proteins quantified in the comparison TAC^Vh^ vs SHAM^Vh^. The first sheet represents the list of all quantified proteins, whereas the second one contains explanations for the column headers.

**Table S5:** List of the phosphorylated peptides quantified in the comparison TAC^Vh^ vs SHAM^Vh^. The first sheet represents the list of all quantified phosphorylation events, whereas the second one contains explanations for the column headers.

**Table S6:** List of the predicted kinases found regulated in the experimental groups. The first sheet lists the predicted kinases in the comparison SHAM^Tr^ vs SHAM^Vh^. The second sheet the predicted kinases in the comparison TAC^Vh^ vs SHAM^Vh^. The third sheet the predicted kinases in the comparison TAC^Tr^ vs TAC^Vh^. The fourth sheet contains explanation for the column headers.

**Table S7:** List of the proteins quantified in the comparison TAC^Tr^ vs TAC^Vh^. The first sheet represents the list of all quantified proteins, whereas the second one contains explanations for the column headers.

**Table S8:** List of the phosphorylated peptides quantified in the comparison TAC^Tr^ vs TAC^Vh^. The first sheet represents the list of all quantified phosphorylated events, whereas the second one contains explanations for the column headers.

**Table S9:** List of the proteins quantified in the comparison SHAM^Tr^ vs SHAM^Vh^. The first sheet represents the list of all quantified proteins, whereas the second one contains explanations for the column headers.

**Table S10:** List of the phosphorylated peptides quantified in the comparison SHAM^Tr^ vs SHAM^Vh^. The first sheet represents the list of all quantified phosphorylation events, whereas the second one contains explanations for the column headers.

**References**

1. Chang YW, Chang YT, Wang Q, Lin JJ, Chen YJ and Chen CC. Quantitative phosphoproteomic study of pressure-overloaded mouse heart reveals dynamin-related protein 1 as a modulator of cardiac hypertrophy. *Mol Cell Proteomics*. 2013;12:3094-107.

2. Cox J and Mann M. MaxQuant enables high peptide identification rates, individualized p.p.b.-range mass accuracies and proteome-wide protein quantification. *Nature Biotechnology*. 2008;26:1367-1372.

3. Dai DF, Hsieh EJ, Chen T, Menendez LG, Basisty NB, Tsai L, Beyer RP, Crispin DA, Shulman NJ, Szeto HH, Tian R, MacCoss MJ and Rabinovitch PS. Global proteomics and pathway analysis of pressure-overload-induced heart failure and its attenuation by mitochondrial-targeted peptides. *Circ Heart Fail*. 2013;6:1067-76.

4. Eid S, Turk S, Volkamer A, Rippmann F and Fulle S. KinMap: a web-based tool for interactive navigation through human kinome data. *BMC Bioinformatics*. 2017;18:16.

5. Gedik N, Krüger M, Thielmann M, Kottenberg E, Skyschally A, Frey UH, Cario E, Peters J, Jakob H, Heusch G and Kleinbongard P. Proteomics/phosphoproteomics of left ventricular biopsies from patients with surgical coronary revascularization and pigs with coronary occlusion/reperfusion: remote ischemic preconditioning. *Scientific reports*. 2017;7:7629-7629.

6. Hamai M, Iwai M, Ide A, Tomochika H, Tomono Y, Mogi M and Horiuchi M. Comparison of inhibitory action of candesartan and enalapril on brain ischemia through inhibition of oxidative stress. *Neuropharmacology*. 2006;51:822-828.

7. Jia D, Zhang CZ, Qiu Y, Chen XF, Jia L, Chen AF, Chai YF, Zhu ZY, Huang J and Zhang C. Cardioprotective mechanisms of salvianic acid A sodium in rats with myocardial infarction based on proteome and transcriptome analysis. *Acta Pharmacol Sin*. 2019;40:1513-1522.

8. Kammers K, Cole RN, Tiengwe C and Ruczinski I. Detecting Significant Changes in Protein Abundance. *EuPA Open Proteom*. 2015;7:11-19.

9. Kanehisa M and Goto S. KEGG: Kyoto Encyclopedia of Genes and Genomes. *Nucleic Acids Research*. 2000;28:27-30.

10. Kanehisa M. Toward understanding the origin and evolution of cellular organisms. *Protein Sci*. 2019;28:1947-1951.

11. Kanehisa M, Furumichi M, Sato Y, Ishiguro-Watanabe M and Tanabe M. KEGG: integrating viruses and cellular organisms. *Nucleic Acids Res*. 2021;49:D545-D551.

12. Kuzmanov U, Guo H, Buchsbaum D, Cosme J, Abbasi C, Isserlin R, Sharma P, Gramolini AO and Emili A. Global phosphoproteomic profiling reveals perturbed signaling in a mouse model of dilated cardiomyopathy. *Proc Natl Acad Sci U S A*. 2016;113:12592-12597.

13. Kuzmanov U, Wang EY, Vanderlaan R, Kim DH, Lee SH, Hadipour-Lakmehsari S, Guo H, Zhao Y, McFadden M, Sharma P, Billia F, Radisic M, Gramolini A and Emili A. Mapping signalling perturbations in myocardial fibrosis via the integrative phosphoproteomic profiling of tissue from diverse sources. *Nat Biomed Eng*. 2020;4:889-900.

14. Lau E, Cao Q, Lam MPY, Wang J, Ng DCM, Bleakley BJ, Lee JM, Liem DA, Wang D, Hermjakob H and Ping P. Integrated omics dissection of proteome dynamics during cardiac remodeling. *Nat Commun*. 2018;9:120.

15. Ledee D, Kang MA, Kajimoto M, Purvine S, Brewer H, Pasa-Tolic L and Portman MA. Quantitative cardiac phosphoproteomics profiling during ischemia-reperfusion in an immature swine model. *Am J Physiol Heart Circ Physiol*. 2017;313:H125-H137.

16. Linscheid N, Logantha S, Poulsen PC, Zhang S, Schrölkamp M, Egerod KL, Thompson JJ, Kitmitto A, Galli G, Humphries MJ, Zhang H, Pers TH, Olsen JV, Boyett M and Lundby A. Quantitative proteomics and single-nucleus transcriptomics of the sinus node elucidates the foundation of cardiac pacemaking. *Nat Commun*. 2019;10:2889.

17. Liu S, Xia Y, Liu X, Wang Y, Chen Z, Xie J, Qian J, Shen H and Yang P. In-depth proteomic profiling of left ventricular tissues in human end-stage dilated cardiomyopathy. *Oncotarget*. 2017;8:48321-48332.

18. Lu D, Xia Y, Chen Z, Chen A, Wu Y, Jia J, Sun A, Zou Y, Qian J and Ge J. Cardiac Proteome Profiling in Ischemic and Dilated Cardiomyopathy Mouse Models. *Front Physiol*. 2019;10:750.

19. Lundby A, Andersen MN, Steffensen AB, Horn H, Kelstrup CD, Francavilla C, Jensen LJ, Schmitt N, Thomsen MB and Olsen JV. In vivo phosphoproteomics analysis reveals the cardiac targets of β-adrenergic receptor signaling. *Sci Signal*. 2013;6:rs11.

20. Lundby A, Rossin EJ, Steffensen AB, Acha MR, Newton-Cheh C, Pfeufer A, Lynch SN, Olesen S-P, Brunak S, Ellinor PT, Jukema JW, Trompet S, Ford I, Macfarlane PW, Krijthe BP, Hofman A, Uitterlinden AG, Stricker BH, Nathoe HM, Spiering W, Daly MJ, Asselbergs FW, van der Harst P, Milan DJ, de Bakker PIW, Lage K, Olsen JV and The QTIIGC. Annotation of loci from genome-wide association studies using tissue-specific quantitative interaction proteomics. *Nature Methods*. 2014;11:868-874.

21. Lundby A, Franciosa G, Emdal KB, Refsgaard JC, Gnosa SP, Bekker-Jensen DB, Secher A, Maurya SR, Paul I, Mendez BL, Kelstrup CD, Francavilla C, Kveiborg M, Montoya G, Jensen LJ and Olsen JV. Oncogenic Mutations Rewire Signaling Pathways by Switching Protein Recruitment to Phosphotyrosine Sites. *Cell*. 2019;179:543-560.e26.

22. Perez-Riverol Y, Csordas A, Bai J, Bernal-Llinares M, Hewapathirana S, Kundu DJ, Inuganti A, Griss J, Mayer G, Eisenacher M, Perez E, Uszkoreit J, Pfeuffer J, Sachsenberg T, Yilmaz S, Tiwary S, Cox J, Audain E, Walzer M, Jarnuczak AF, Ternent T, Brazma A and Vizcaino JA. The PRIDE database and related tools and resources in 2019: improving support for quantification data. *Nucleic Acids Res*. 2019;47:D442-D450.

23. Rath S, Sharma R, Gupta R, Ast T, Chan C, Durham TJ, Goodman RP, Grabarek Z, Haas ME, Hung WHW, Joshi PR, Jourdain AA, Kim SH, Kotrys AV, Lam SS, McCoy JG, Meisel JD, Miranda M, Panda A, Patgiri A, Rogers R, Sadre S, Shah H, Skinner OS, To TL, Walker MA, Wang H, Ward PS, Wengrod J, Yuan CC, Calvo SE and Mootha VK. MitoCarta3.0: an updated mitochondrial proteome now with sub-organelle localization and pathway annotations. *Nucleic Acids Res*. 2021;49:D1541-d1547.

24. Raudvere U, Kolberg L, Kuzmin I, Arak T, Adler P, Peterson H and Vilo J. g:Profiler: a web server for functional enrichment analysis and conversions of gene lists (2019 update). *Nucleic Acids Research*. 2019;47:W191-W198.

25. Speerschneider T, Grubb S, Metoska A, Olesen SP, Calloe K and Thomsen MB. Development of heart failure is independent of K+ channel-interacting protein 2 expression. *J Physiol*. 2013;591:5923-37.

26. Su Z, Zhu H, Zhang M, Wang L, He H, Jiang S, Hou FF and Li A. Salt-induced changes in cardiac phosphoproteome in a rat model of chronic renal failure. *PLoS One*. 2014;9:e100331.

27. Thomsen MB, Nielsen MS, Aarup A, Bisgaard LS and Pedersen TX. Uremia increases QRS duration after β-adrenergic stimulation in mice. *Physiol Rep*. 2018;6:e13720.

28. Wang X, Shen X, Weil BR, Young RF, Canty JM and Qu J. Quantitative proteomic and phosphoproteomic profiling of ischemic myocardial stunning in swine. *Am J Physiol Heart Circ Physiol*. 2020;318:H1256-H1271.

29. Wiredja DD, Koyutürk M and Chance MR. The KSEA App: a web-based tool for kinase activity inference from quantitative phosphoproteomics. *Bioinformatics*. 2017;33:3489-3491.

30. Zhan DY, Morimoto S, Du CK, Wang YY, Lu QW, Tanaka A, Ide T, Miwa Y, Takahashi-Yanaga F and Sasaguri T. Therapeutic effect of {beta}-adrenoceptor blockers using a mouse model of dilated cardiomyopathy with a troponin mutation. *Cardiovasc Res*. 2009;84:64-71.
